# Interpolation-split: a data-centric deep learning approach with big interpolated data to boost airway segmentation performance

**DOI:** 10.1186/s40537-024-00974-x

**Published:** 2024-08-04

**Authors:** Wing Keung Cheung, Ashkan Pakzad, Nesrin Mogulkoc, Sarah Helen Needleman, Bojidar Rangelov, Eyjolfur Gudmundsson, An Zhao, Mariam Abbas, Davina McLaverty, Dimitrios Asimakopoulos, Robert Chapman, Recep Savas, Sam M. Janes, Yipeng Hu, Daniel C. Alexander, John R. Hurst, Joseph Jacob

**Affiliations:** 1 Satsuma Lab, Centre for Medical Image Computing, University College London, 1st Floor, 90 High Holborn, London, WC1V6LJ UK; 2 Department of Computer Science, University College London, London, UK; 3 Department of Medical Physics and Biomedical Engineering, University College London, London, UK; 4 Department of Respiratory Medicine, Ege University Hospital, Izmir, Turkey; 5 Medical School, University College London, London, UK; 6 School of Clinical Medicine, University of Cambridge, Cambridge, UK; 7 Interstitial Lung Disease Service, Department of Respiratory Medicine, University College London Hospitals NHS Foundation Trust, London, UK; 8 Department of Radiology, Ege University Hospital, Izmir, Turkey; 9 Lungs for Living Research Centre, UCL, London, UK; 10 UCL Respiratory, University College London, London, UK; 11 Respiratory Medicine, Royal Free London NHS Foundation Trust, London, UK

## Abstract

The morphology and distribution of airway tree abnormalities enable diagnosis and disease characterisation across a variety of chronic respiratory conditions. In this regard, airway segmentation plays a critical role in the production of the outline of the entire airway tree to enable estimation of disease extent and severity. Furthermore, the segmentation of a complete airway tree is challenging as the intensity, scale/size and shape of airway segments and their walls change across generations. The existing classical techniques either provide an undersegmented or oversegmented airway tree, and manual intervention is required for optimal airway tree segmentation. The recent development of deep learning methods provides a fully automatic way of segmenting airway trees; however, these methods usually require high GPU memory usage and are difficult to implement in low computational resource environments. Therefore, in this study, we propose a data-centric deep learning technique with big interpolated data, Interpolation-Split, to boost the segmentation performance of the airway tree. The proposed technique utilises interpolation and image split to improve data usefulness and quality. Then, an ensemble learning strategy is implemented to aggregate the segmented airway segments at different scales. In terms of average segmentation performance (dice similarity coefficient, DSC), our method (A) achieves 90.55%, 89.52%, and 85.80%; (B) outperforms the baseline models by 2.89%, 3.86%, and 3.87% on average; and (C) produces maximum segmentation performance gain by 14.11%, 9.28%, and 12.70% for individual cases when (1) nnU-Net with instant normalisation and leaky ReLU; (2) nnU-Net with batch normalisation and ReLU; and (3) modified dilated U-Net are used respectively. Our proposed method outperformed the state-of-the-art airway segmentation approaches. Furthermore, our proposed technique has low RAM and GPU memory usage, and it is GPU memory-efficient and highly flexible, enabling it to be deployed on any 2D deep learning model.

## Introduction

Abnormal dilatation of the airways is a key feature in the diagnosis of idiopathic pulmonary fibrosis (IPF) patients. Disease extent and severity in IPF can be assessed by the visual analysis of high-resolution CT images by radiologists. This approach, however, is subjective and time-consuming. Automated airway tree analysis [1, 2] is an alternative method that enables an objective quantitative assessment of airway damage and disease severity in IPF. The key component of airway tree analysis is establishing the 3D geometry of the airway tree, and the standard approach to obtaining the airway tree is image segmentation.

Airway segmentation is an active research area [3]. The goal is to produce a complete airway tree, including the trachea, bronchi, bronchioles, and terminal bronchioles. The segmentation task is challenging as the intensity, scale/size, and shape of airway segments and their walls change across generations. Classical segmentation methods such as the Frangi filter [4, 5] and the region-growing method [6] were first used to segment the airway tree. The Frangi enhancement filter constructs a Hessian matrix to extract tubular-like tissues (i.e., airways) and remove non-tubular tissues (i.e., lung). This approach shows promise for airway segmentation. However, the segmented airway tree is limited to the first few branching airway generations (i.e., between the 1st and 6th generations). Furthermore, it requires tuning the parameters (α, β and σ) manually for extracting the optimal airway tree. This process is time-consuming and not user-friendly for clinicians. Employing a region-growing algorithm is another approach to segmenting the airway tree. A seed point is first placed at the trachea, then the region is grown by adding neighbouring voxels with a predefined intensity. The algorithm stops when no more voxels can be added. There are several drawbacks to this approach. Intensity thresholding is used to select voxels, but it causes leakage (over-segmentation) when an aggressive threshold is used. Conversely, the airway is undersegmented when a conservative threshold is used. Therefore, the completeness of the airway tree produced by this approach is limited.

Recent advances in deep learning provide new opportunities for segmentation. It utilises data and GPU technology and offers a fast and fully automatic method to perform segmentation. Deep learning (DL) can be divided into two branches: (1) model-centric and (2) data-centric. Model-centric deep learning focuses on the model architecture and keeps the data unchanged. Popular models have been developed to tackle the segmentation challenge. For example, SegNet [7] and HRNet [8] are proposed for general segmentation. U-Net [9] and V-Net [10] are deployed for medical image segmentation. These models produce good segmentation, though they require high GPU memory usage. On the other hand, data-centric deep learning focuses on the data and keeps the model unchanged. Data augmentation [11] is an example of manipulating the source data to produce more varied samples. It uses geometrical transformations (i.e., flip, rotate, and crop) to modify the images. The model's performance can be improved by training on a dataset with richer features. Active learning [12] is another example of a data-centric technique. It aims to select the most useful data for labelling and permits the user to interact with the deep learning model to complete the data annotation. This technique improves the efficiency of the annotation task. Furthermore, a data-centric deep learning approach is particularly attractive as it requires low GPU memory usage and is straightforward to implement.

Interpolation has been widely used in image processing. The mechanism of interpolation involves resampling; several interpolating functions have been used for image resampling [13], i.e., the nearest neighbour function, the linear function, and the cubic B-spline function. Interpolation has also been used in image augmentation [14]. It is applied to either input space or feature space. The purpose of this technique is to diversify the training samples by manipulating features in the input or feature spaces and, hence, improve the generalisation. Furthermore, it can be used to fill in the blank part of the image after image manipulation [15], i.e., rotation. Cropping is used in conjunction with interpolation to achieve the desired results. Existing techniques such as random scaling, random cropping, and random cropping with scaling can increase the variability of the training images. For example, use RandomResizedCrop from PyTorch. It crops the image randomly, and the sub-image is subsequently upscaled to the original image size by interpolation. The drawback of this approach is that the random cropping can miss the important features in the image, and the up-scaling can increase the blurring and edge effects on the sub-image. To resolve these issues, a novel technique, Interpolation-Split, is proposed in this study. It performs systematic up-scaling, followed by systematic splitting on the image. In the context of airway segmentation, this new approach can ensure all airways are captured and utilised. Further, it minimises blurring and edge effects when interpolation is performed.

Additionally, no study focuses on a purely data-centric approach for airway segmentation. Therefore, in this study, we propose a 2D data-centric deep learning method for the automated segmentation of airway trees on HRCT images. The proposed technique is evaluated by comparing the segmentation performance with three baseline models: 2D nnU-Net with instant normalisation (IN) plus leaky ReLU, 2D nnU-Net with batch normalisation (BN) plus ReLU, and 2D modified dilated U-Net.

The main contributions of this study are:

- The first study to propose a 2D data-centric deep learning method with interpolation that segments the airways on HRCT images.
- The proposed technique utilises interpolation and image split to improve data usefulness and quality.
- The study combines big interpolated data (972,655 samples) and a data-centric deep learning method to boost airway segmentation performance.
- An ensemble learning strategy is implemented to aggregate the segmented airway segments at different scales.
- The proposed technique has low RAM and GPU memory usage, is GPU memory-efficient, and is highly flexible to be deployed in any 2D deep learning model.

The organisation of the rest of the paper is structured as follows: Section II reviews the latest and relevant research work regarding airway segmentation. The methods and methodology of the proposed work are presented in Section III. The computational results are shown in Section IV. Section V discusses the research findings and addresses the potential implications, limitations, and future research directions. Finally, Section VI summarises and concludes the key findings, contribution, and potential impact of the proposed work.

## Related work

The studies related to model-centric deep learning in airway segmentation are summarised below. A convolutional neural network (CNN)-based leak detection method to improve airway segmentation was proposed by Charbonnier et al. [16]. Yun et al. [17] presented a 2.5D CNN for airway segmentation. This approach achieved about 90% DSC accuracy. A 3D U-Net to detect topological leaks was employed by Nadeem et al. [18]. The intensity threshold was adjusted on the probability map, and a freeze-and-growth algorithm was used to correct the leaks. Qin et al. [19] developed a simple-yet-effective deep learning method for this task. It utilised a context-scale fusion strategy to improve the connectivity between airway segments. The DSC of this approach is 93% on a public dataset. A three-dimensional multi-scale feature aggregation network was proposed by Zhou et al. [20] to handle the difference in scale of substructures during airway tree segmentation. This method produced results with 86.18% DSC and 79.31% true positive rate (TPR). Further, a simple and low-memory 3D U-Net was developed by Garcia‑Uceda et al. [21]. It processed large 3D image patches in a single pass within the network, creating a robust and efficient analysis. Zheng et al. [22] proposed WingsNet with group supervision to deal with class imbalances between airway and non-airway regions. They identified the gradient erosion and dilation problem and designed a group supervision to enhance the training of the network. A general union loss was also developed to tackle the intra-class imbalance issue through distance-based weights and element-wise focus on the hard-to-segment regions. The branch detection rate of the proposed method is 80.5%. A coarse-to-fine segmentation framework was deployed by Guo et al. [23]. It utilised a multi-information fusion convolution neural network (Mif-CNN) and a CNN-based region growing for main airway and small branch segmentation. The DSCs of this work were 93.5% and 95.8% for private and public datasets respectively. Wang et al. [24] developed a spatially fully connected tubular network with a novel radial distance loss for 3D tubular-structure segmentation. The method provided better airway tree segmentation than the baseline U-Net model. A joint 3D U-Net-Graph Neural Network-based method was presented by Juarez et al. [25]. It used graph convolutions to improve airway connectivity. Wu et al. [26] proposed a long-term slice propagation method for airway segmentation. The method achieved 92.95% DSC. A novel label refinement method was developed by Chen et al. [27] to correct the structural errors in airway segmentation. It produced airway segmentation with DSC between 79 and 81%. Wang et al. [28] proposed NaviAirway, which finds finer bronchioles with a bronchiole-sensitive loss function and a human-vision-inspired iterative training strategy. Zhao et al. [29] developed Group Deep Dense Supervision for small bronchiole segmentation. This method has a high sensitivity for detecting fine-scale branches and outperforms state-of-the-art methods by a large margin (+ 12.8% in branch detection and + 8.8% in tree detection). More recently, Weng et al. [30] developed a post-processing approach that leverages a data-driven method to repair the topology of disconnected pulmonary tubular structures (i.e., airways). Wang et al. [31] proposed an anatomy-aware multi-class airway segmentation method enhanced by topology-guided iterative self-learning. A semi-supervised pulmonary airway segmentation with a two-stage feature specialisation mechanism was presented by Gu et al. [32]. Yu et al. [33] proposed AirwayFormer that uses the latent relationships within the tree structure and airway nomenclature for airway segmentation and labeling. Støverud et al. [34] introduced a airway segmentation benchmark dataset with challenging pathology and presented a multiscale fusion design for automatic airway segmentation. Hu et al. [35] developed a large-kernel attention network with distance regression and topological self-correction for airway segmentation. Their methods achieved superior performance on BAS and ATM22 Challenge datasets. Carmo et al. [36] developed an end-to-end segmentation method (MEDPSeg) for pulmonary structures and lesions in CT images. The method utilised hierarchical polymorphic multitask learning and outperformed several existing methods. A connectivity-aware pulmonary airway segmentation was proposed by Zhang et al. [37]. It includes a connectivity-aware surrogate module that balances the training progress within-class distribution and a local-sensitive distance module that identifies the breakage and minimises the variation of the distance map between the prediction and ground-truth. Yuan et al. [38] proposed an end-to-end multi-scale airway segmentation framework based on pulmonary CT images. It employed a 2D full-airway SegNet (2D FA-SegNet) and 3D airway RefineNet to improve the airway segmentation. Their proposed method showed the highest DSC of 0.931. Zhao et al. [39] presented a skeleton-level annotation (SkA) method tailored to the airway, which simplifies the annotation workflow while enhancing annotation consistency and accuracy, preserving the complete topology. Furthermore, a skeleton-supervised learning framework was proposed to achieve accurate airway segmentation. To summarise the literature review, we provide a summary of the latest state-of-the-art approaches with their advantages and challenges for airway segmentation in Table 1.

**Table 1:** Summary of the latest state-of-the-art approaches with advantages and challenges

References	Techniques	Advantages	Challenges
Weng et al. [30]	A two-channel 3D neural network that efficiently identifies key points and bridges disconnected components	Utilises a training data synthesis pipeline that generates disconnected data from complete pulmonary structures	Difficult to handle multiple disconnected components, i.e., distinguishing between airways, arteries and veins
Wang et al. [31]	An anatomy-aware multi-class airway segmentation method enhanced by topology-guided iterative self-learning	(1) Handles the severe intra-class imbalance of the airway (2) Introduces a breakage attention map and design a topology guided pseudo-label refinement method by iteratively connecting breaking branches commonly existed from initial pseudo-labels (3) Low GPU memory	Inaccurate prediction of airways in scans due to the structure not being visible in low resolution CT
Gu et al. [32]	A semi-supervised pulmonary airway segmentation with two-stage feature specialization mechanism	Requires much fewer ground truth airway labels	Fails to detect the smallest branches deep inside the lungs
Yu et al. [33]	AirwayFormer: Structure-Aware Boundary-Adaptive Transformers for Airway Anatomical Labeling	(1) Accurate airway labeling up to subsegmental level by exploiting the latent structural relationships (2) A weight generator is designed to mitigate the overfitting caused by individual variation via adaptive decision boundary Adjustment	(1) Fails to detect the smallest branches deep inside the lungs (2) High GPU memory usage
Støverud et al. [34]	An airway segmentation benchmark dataset with challenging pathology	(1) Release of a new public benchmark dataset called AeroPath, comprising of 27 annotated contrast CT scans with severe pathologies including tumors of various size for SOTA method development (2) Proposes a multi-scale fusion design for improving airway segmentation	(1) Fails to detect the smallest branches deep inside the lungs (2) High GPU memory usage
Hu et al. [35]	A large-kernel attention network with distance regression and topological self-correction for airway segmentation	(1) Improves airway segmentation by topological learning and customized loss functions for thin branches (2) introduces self-correction loss to reduce false positive rate	High GPU memory usage
Carmo et al. [36]	A hierarchical polymorphic multitask learning framework for the segmentation of ground-glass opacities, consolidation, and pulmonary structures on computed tomography	Demonstrates impressive adaptability to heterogeneous unseen scans from multiple sources, fields of view, resolution, and other parameters, producing results similar to specialised methods from each target	Inaccurate prediction of vessels and airways in scans with high distance between slices due to these structures not being visible in low resolution CT
Zhang et al. [37]	A connectivity-aware segmentation framework	Improves the connectivity via balancing the recall/precision metrics and reducing the breakages of small bronchi	(1) High sensitivity of small bronchi will lead to the over- segmentation of airway (2) High GPU memory usage
Yuan et al. [38]	A two-stage and 2D plus 3D framework for multi-scale airway tree segmentation	(1) 2D FA-SegNet for full airway tree segmentation (2) 3D ARNet for intrapulmonary trachea refinement and focuses more on the small bronchioles in the lung	High GPU memory usage
Zhao et al. [39]	A skeleton-level annotation (SkA) method tailored to the airway	The method (1) simplifies the annotation workflow (2) enhancing annotation consistency and accuracy (3) preserves the complete topology	(1) Sensitive to noisy airway labels (2) Potential bias to a single dataset for training

## Methods and methodology

### Clinical data

The clinical data (n = 30) contained healthy subjects, patients with heart disease, and patients with IPF. It included a healthy subject and six patients with heart disease or IPF from the EXACT09 dataset [40], six healthy, never-smoking subjects, and 17 IPF patients from University College London Hospital. The study was carried out in accordance with the recommendations of University College London Research Ethics Committee, with written informed consent from all subjects. The data including source images and their ground-truth masks, were further divided into training (66%) and validation (34%) sets. Table 2 shows the subject/patient information in the validation set. The number of samples (source images) for training and validation is shown in Table 3.

**Table 2:** The subject/patient information in the validation set

Subject/patient	Status
case 1	Healthy
case 2	Healthy
case 3	Patient with IPF
case 4	Patient with heart disease
case 5	Patient with IPF
case 6	Patient with IPF
case 7	Healthy
case 8	Patient with IPF
case 9	Patient with IPF
case 10	Patient with heart disease

**Table 3:** The number of samples (source images) for training and validation

Interpolation ratio (*ir*)	Training set	Validation set
1 (original dataset)	7552	3891
2	30,208	15,564
4	120,832	62,256
8	483,328	249,024
Total	641,920	330,735

### Data pre-processing

The data were preprocessed in three steps: (1) ImageJ was used to convert the source images from DICOM format to TIFF format. (2) The images were subsequently normalised by using the following settings to emphasise lung tissue visualisation: W = 1500 HU, L = -500 HU. (3) The intensity of the normalised images was rescaled in the range 0 to 255 HU. The annotation of the ground-truth mask was performed on a 3D Slicer.

### The overview of the proposed method

**Fig. 1:**
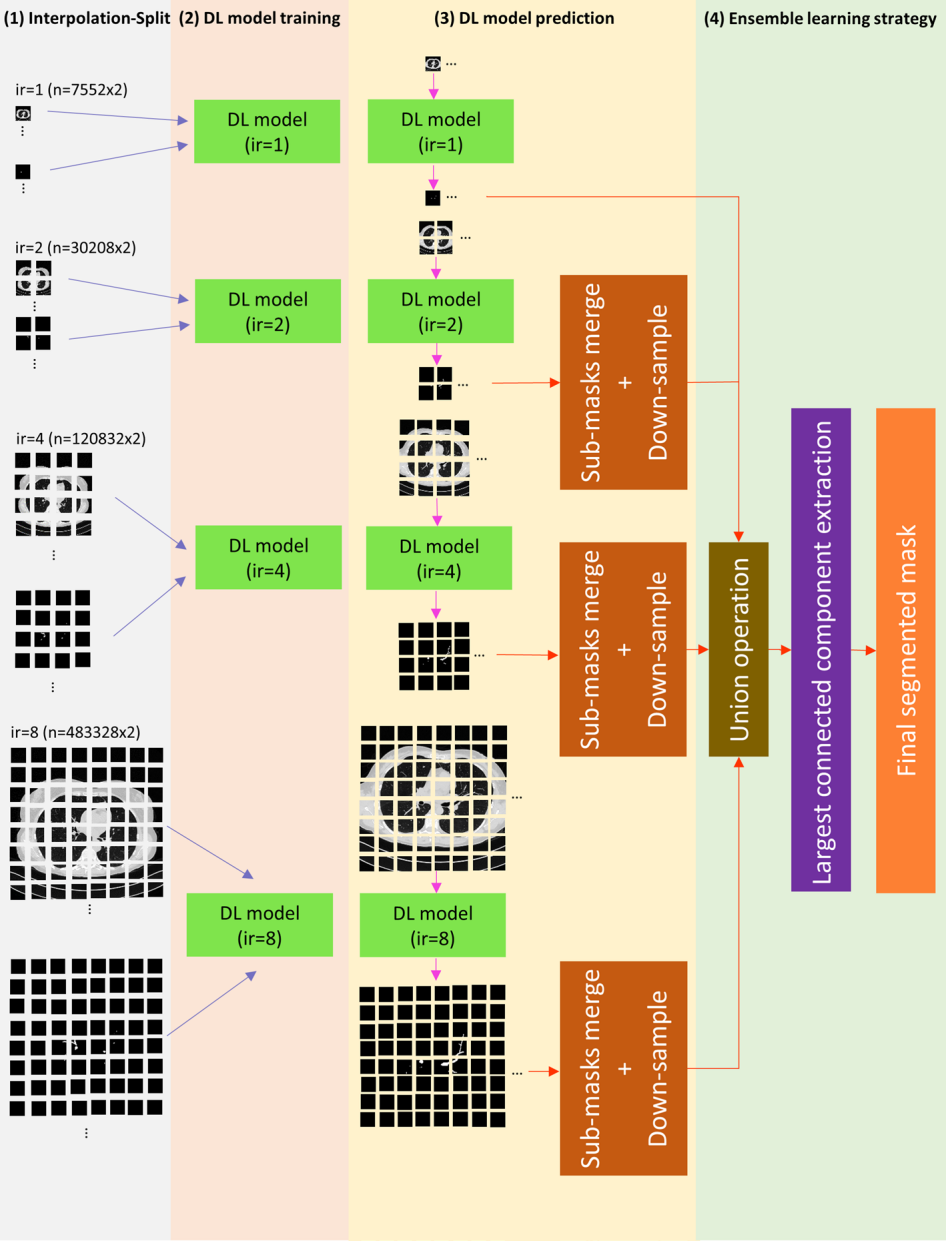
The overview of our proposed method

The overview of our proposed method is shown in Fig. 1. It is comprised of four main components: (1) Interpolation-Split (2) Deep learning model training (3) Deep learning model prediction (4) Ensemble learning strategy. The details of each component are described in the following paragraphs.

### Interpolation-Split

#### The algorithm and the workflow

**Fig. 2:**
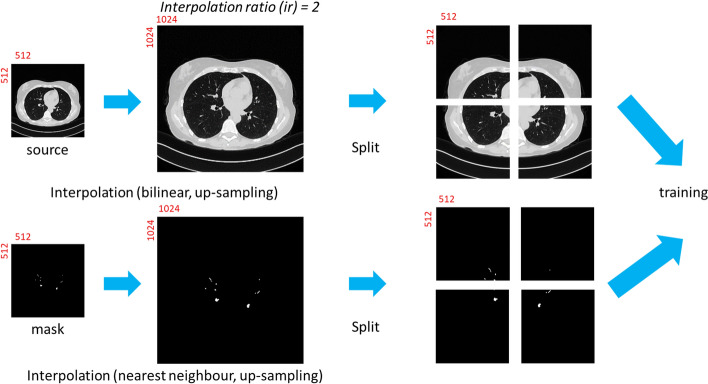
The interpolation and split mechanism


The algorithm of Interpolation-Split is shown below, while the workflow and the details of Interpolation-Split are as follows: The CT image and its mask are zoomed in at various scales. The zoomed-in CT images and masks are produced by interpolation and split. The original CT images are up-sampled by bi-linear interpolation, while the original masks are up-sampled by nearest neighbour interpolation. Then, the interpolated image is split into sub-images with fixed dimensions (512 × 512). Here, an interpolation ratio (*ir*) is defined to control the zoom-in scale. For example, the dimension of the interpolated image (1024 × 1024) is doubled from the original image (512 × 512) when *ir* is set to 2. Then, the interpolated image is split into four sub-images (512 × 512). The interpolation and split mechanism (i.e., ir2) is demonstrated in Fig. 2. Further, the effect of the interpolated ratio (*ir* = 2, 4, and 8) is investigated. It should be noted that no interpolation and split is performed for *ir* = 1.

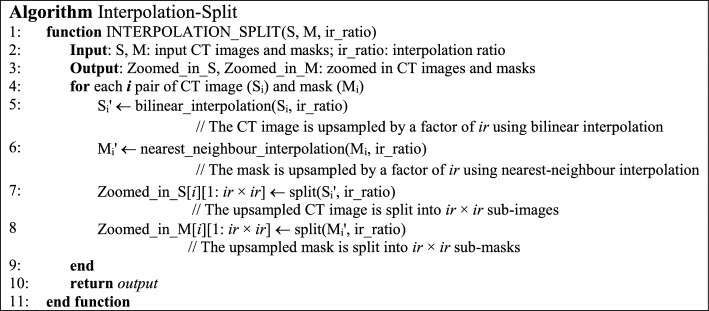


The pseudo-code of Interpolation-Split.

#### Mathematical model and numerical analysis

A mathematical model is introduced to study the fundamental properties of our proposed method. The focus is on the interpolation of CT images, as the existing technique could add artefacts to the upsampled CT images. In our proposed work, bilinear interpolation is employed to upsample the CT images; it can be treated as linear interpolation in the lateral direction, followed by linear interpolation in the axial direction. Therefore, bilinear interpolation is our mathematical model. It can be expressed as follows:

The objective is to estimate unknown point *f(S)* given four known points, *f(P*_*11*_*)*, *f(P*_*21*_*)*, *f(P*_*12*_*),* and *f(P*_*22*_*)*.

1 $$f\left(S\right)=f\left(x,y\right)=\frac{{y}_{2}-y}{{y}_{2}-{y}_{1}}f\left(x,{y}_{1}\right)+ \frac{y-{y}_{1}}{{y}_{2}-{y}_{1}}f\left(x,{y}_{2}\right)$$

where

2 $$f\left(x,{y}_{1}\right)=f\left({C}_{1}\right)= \frac{{x}_{2}-x}{{x}_{2}-{x}_{1}}f\left({P}_{11}\right)+\frac{x-{x}_{1}}{{x}_{2}-{x}_{1}}f\left({P}_{21}\right)$$

3 $$f\left(x,{y}_{2}\right)=f\left({C}_{2}\right)= \frac{{x}_{2}-x}{{x}_{2}-{x}_{1}}f\left({P}_{12}\right)+\frac{x-{x}_{1}}{{x}_{2}-{x}_{1}}f\left({P}_{22}\right)$$

**Fig. 3:**
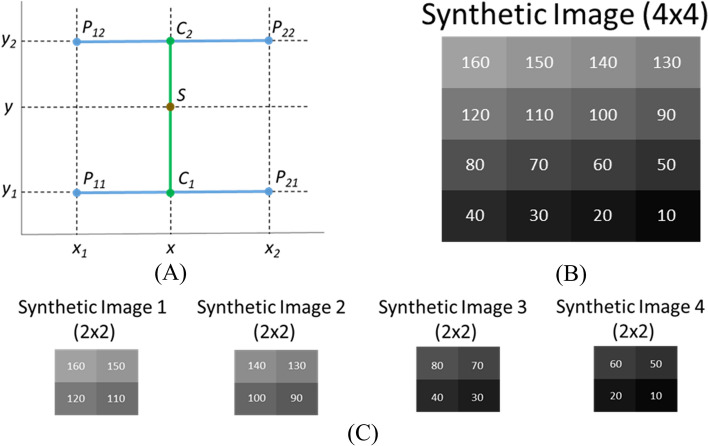
**A** The coordinate system of bilinear interpolation **B** Synthetic image (4 × 4) **C** Four synthetic images (2 × 2)

The coordinates of points *S*, *C*_*1*_, *C*_*2*_, *P*_*11*_, *P*_*21*_, *P*_*12*_ and *P*_*22*_ are shown in Fig. 3A.

A 4 × 4 synthetic image (Fig. 3B) was used to numerically investigate the intensity change between the existing technique and the proposed method. (1) Existing technique: the 4 × 4 synthetic image was split into four 2 × 2 synthetic images (Fig. 3C), and then each 2 × 2 synthetic image was upsampled to 4 × 4 image by using the mathematical model. Finally, the upsampled (4 × 4) images were merged together to form an 8 × 8 grayscale image. (2) Interpolation-Split: the 4 × 4 simulated image was upsampled by a factor of two using the mathematical model. An 8 × 8 grayscale image was produced. The intensity across the lateral and axial directions was analysed for both images.

#### Blurring and edge effects

The blurring and edge effects in CT images were investigated and compared between the existing technique and Interpolation-Split. A slice was selected from each case, and then a set of sub-images was created using the existing technique and Interpolation-Split. (1) Existing technique: a single image (512 × 512) was cropped into 64 sub-images (8 × 8), then each sub-image was up-scaled to the original size (512 × 512). (2) Interpolation-Split: a single image (512 × 512) was up-scaled to 4096 × 4096 (ir8), then the interpolated image was split into 64 sub-images (512 × 512). Four sub-images from each case were selected for comparison. In total, 120 paired sub-images were produced. Diagonal Laplacian [41] was employed to measure the sharpness of the sub-image. Further, a paired *t*-test was used to evaluate whether there is any statistical significance in sharpness between the sub-images produced by the existing technique and Interpolation-Split. A *p*-value < 0.05 was considered significant for statistical analysis. The analysis was performed on SPSS (version 27, IBM).

### Selected models for performance evaluation

Three state-of-the-art models were selected for evaluating our proposed method. These 2D models are (A) nnU-Net with instant normalisation and leaky ReLU; (B) nnU-Net with batch normalisation and ReLU; and (C) modified dilated U-Net.

#### nnU-Net

nnU-Net [42] is a deep learning based semantic segmentation method. It offers automatic configuration including pre-processing, network architecture, training and post-processing for any segmentation task. In this study, two network configurations—instant normalisation with leaky ReLU and batch normalisation with ReLU—were chosen to evaluate our Interpolation-Split method.

#### Modified dilated U-Net

**Fig. 4:**
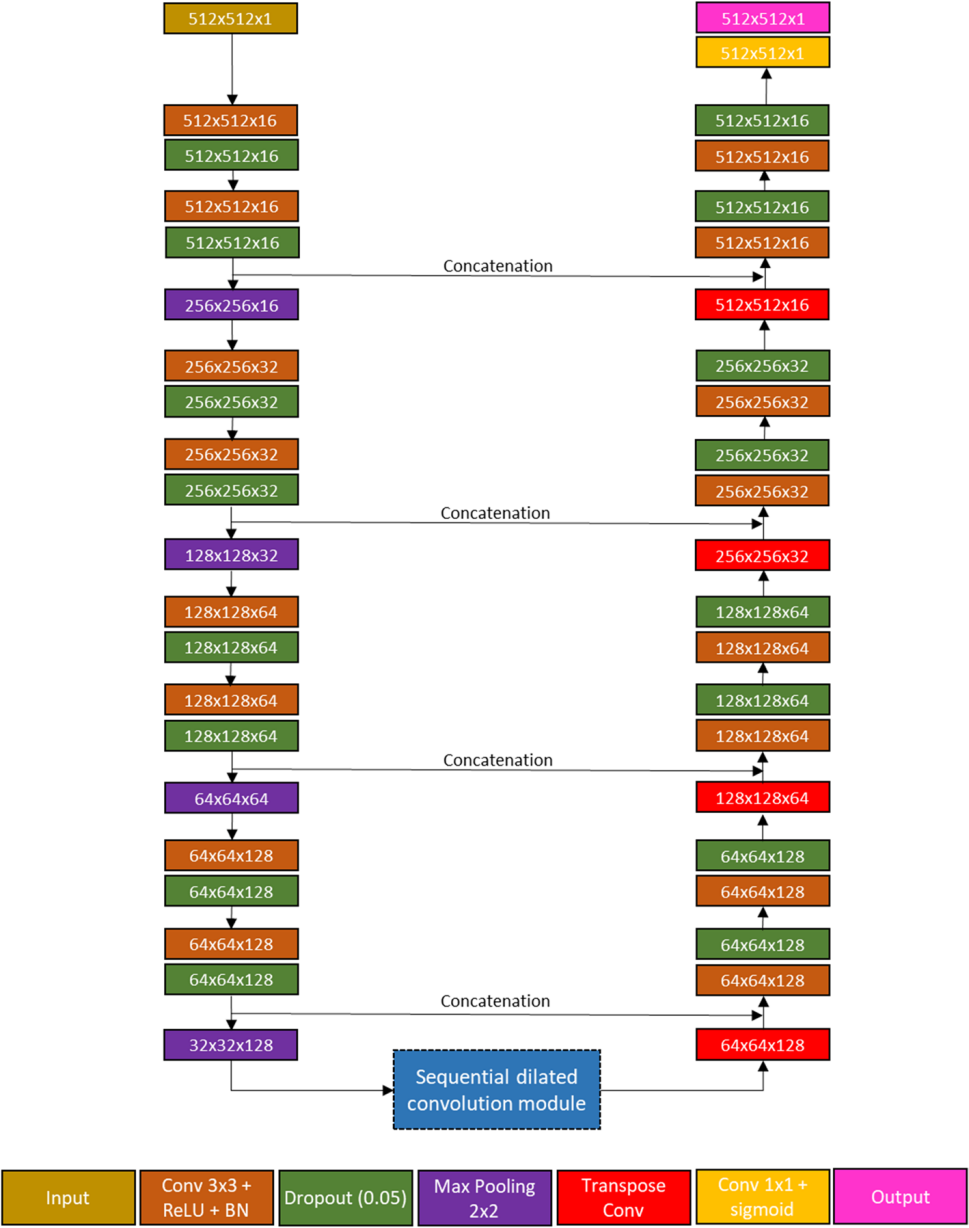
The network architecture of the modified dilated U-Net

**Fig. 5:**
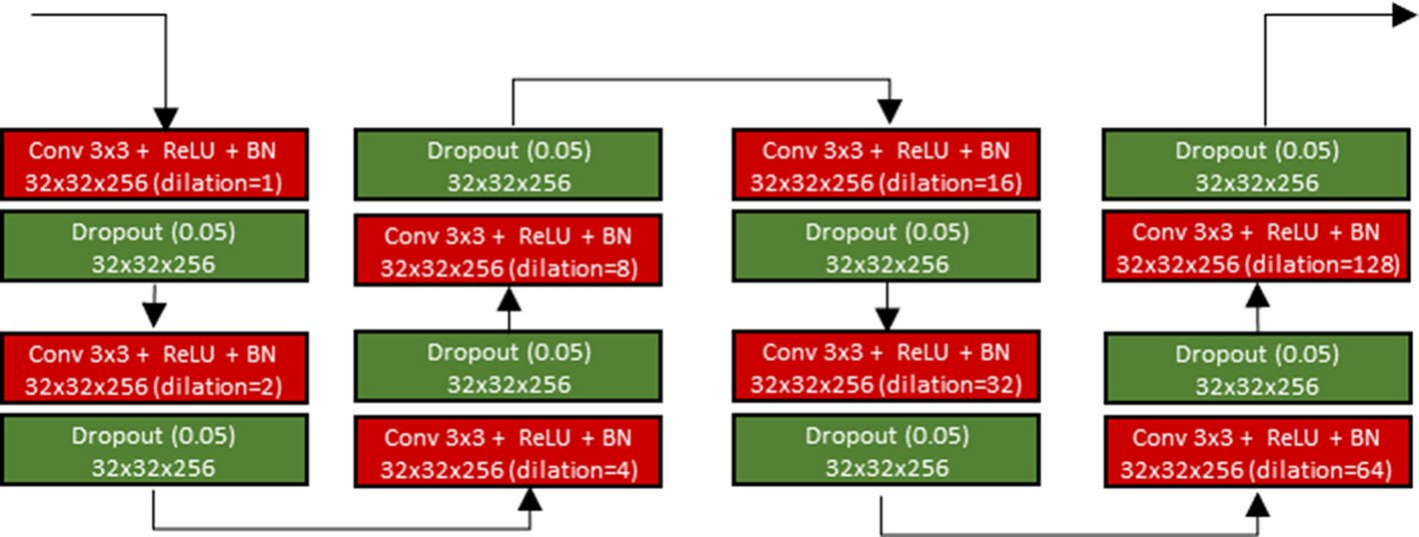
The sequential dilated convolution module

The airway was segmented using a modified dilated U-Net. A dilated U-Net is an extended model of the original U-Net [9] and adopts an encoder-decoder architecture. The encoding path captures features from images, and the decoding path localises these features. A sequential dilation module [43] is employed in the bottleneck layer, and this improves global context capture and maintains the resolution of the feature map. Furthermore, the dilated U-Net was modified by introducing batch normalisation and dropout. These modifications improve the model's stability and segmentation performance. The schematic diagram of the modified dilated U-Net and the sequential dilation module are shown in Figs. 4 and 5.

### Deep learning model training and implementation

The proposed models (per *ir*) were trained and implemented on a high-performance cluster with deep learning frameworks installed. Specifically, PyTorch (v2.0.1), Tensorflow (v1.1.4), and Keras (v2.2.4) were executed on Linux (Rocks 7.0/CentOS 7.9.2009). Furthermore, various computing machines with Intel/AMD multi-core CPU chipsets and Nvidia GPU cards were used to complete the training.

nnU-Net provided an automatic configuration for model training. The configuration includes fixed, rule-based, and empirical parameters. The setting of fixed parameters is shown in Table 4.

**Table 4:** The setting of fixed parameters for models (nnU-Net) training

SGD with Nesterov momentum (*μ* = 0.99) optimiser	
Learning rate (Poly learning rate schedule, initial)	0.01
Epochs	1000 epochs × 250 mini-batches

The trained models for modified dilated U-Net were produced by employing the Adam optimiser, ReduceLROnPlateau, and early stopping. The setting of parameters is shown in Table 5.

**Table 5:** The setting of parameters for models (modified Dilated U-Net) training

Adam optimiser	
Learning rate (initial)	10^–3^
Epochs	200
ReduceLROnPlateau	
Factor	10^–1^
Patience	3
Min_lr	10^–5^
Early stopping	
Patience	10

### Deep learning model prediction

**Fig. 6:**
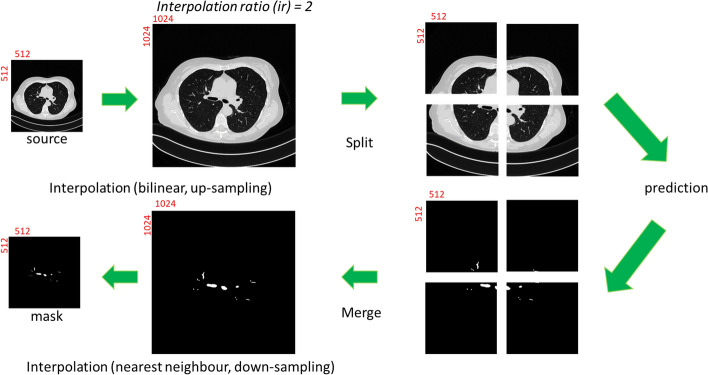
The workflow of prediction mechanism

The prediction (per *ir*) was done using the trained models above. The unseen source images were interpolated and split to form the inputs for model prediction. When the prediction was complete, the initial predicted masks were merged and down-sampled (nearest neighbour) to the final mask with size 512 × 512. The workflow of the prediction mechanism (i.e., ir2) is shown in Fig. 6.

### The loss function and the evaluation metric

The loss function, combined loss, was used to train the deep learning model. The combined loss function includes binary cross entropy (BCE) loss and dice similarity coefficient (DSC) loss. The BCE is used to calculate the difference between the two probability distributions (foreground vs. background), while the DSC is used to measure the similarity between predicted segmentation and ground-truth segmentation. It should be noted that DSC is also employed to evaluate the segmentation performance. Mathematically, the loss function and the evaluation metric can be represented by the following equations (Eqs. 4–7).

4 $$BCE \,loss=-\frac{1}{n}{\sum }_{i=0}^{n}({(y}_{i}\times {log \widehat{y}}_{i}) +\left(1-{y}_{i}\right)\times \mathit{log}\left(1-{\widehat{y}}_{i}\right))$$

5 $$DSC=\left(2 \times \frac{\left|y\cap \widehat{y}\right|}{\left|y\right|+\left|\widehat{y}\right|}\right)= \frac{2{\sum }_{i=0}^{n}({y}_{i} \times {\widehat{y}}_{i})}{{\sum }_{i=0}^{n}{y}_{i} + {\sum }_{i=0}^{n}{\widehat{y}}_{i}}$$

6 $$DSC\, loss=1- DSC=1-\left(\frac{2{\sum }_{i=0}^{n}{(y}_{i} \times {\widehat{y}}_{i})}{{\sum }_{i=0}^{n}{y}_{i} + {\sum }_{i=0}^{n}{\widehat{y}}_{i}}\right)$$

7 $$Combined \,Loss=(0.5 \times BCE\, loss) +(0.5 \times DSC\, loss)$$

where *y* is the ground-truth label, $\widehat{y}$ is the predicted mask, and *n* is the total number of pixels.

### Ensemble learning strategy

The baseline model (ir1) has the ability to segment the airway from the trachea to 6–8 airway generations, while those 9 or above airway generations are missed. An ensemble learning strategy is proposed to overcome the segmentation limitation. By increasing *ir* (i.e., ir2, ir4, and ir8), the optimal segmented airway is shifted towards the airway with a smaller diameter or higher generation. Then, the optimally segmented airways with various *ir* are aggregated. Finally, the airway tree with higher generations (9 or above) is produced.

**Fig. 7:**
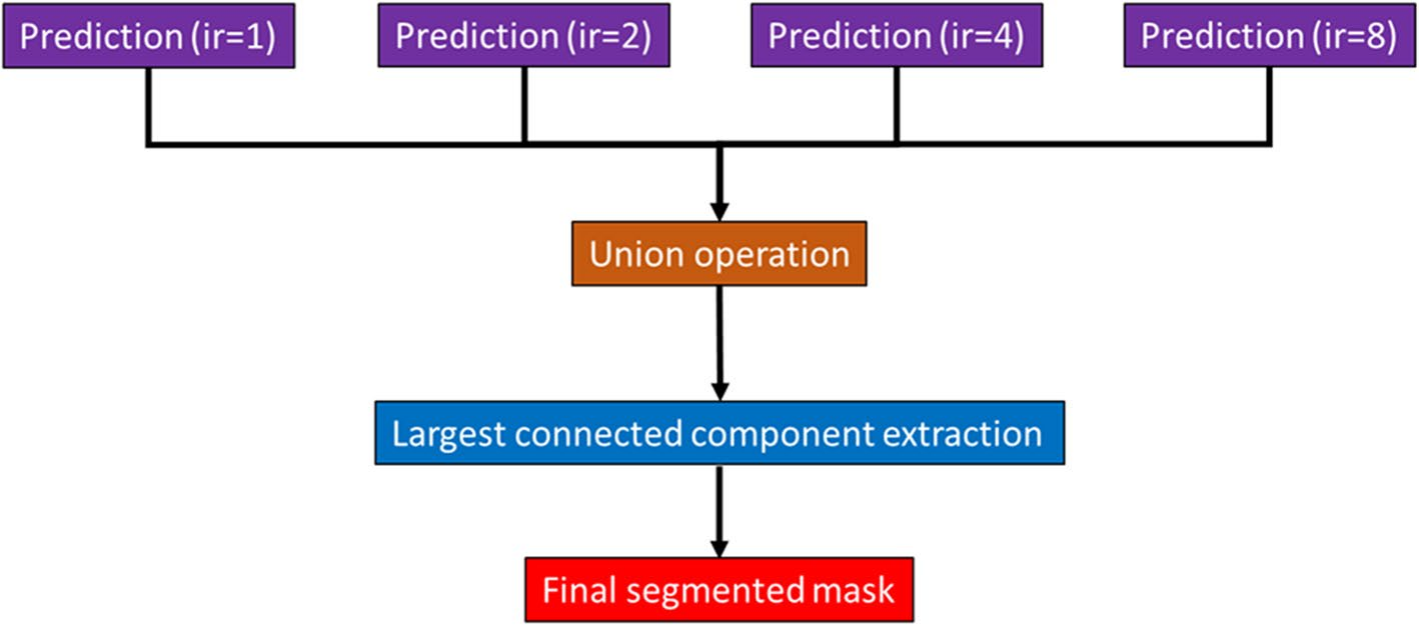
The workflow of ensemble learning strategy

The segmented masks from ir = 1, 2, 4, and 8 are aggregated to form a combined mask. This is done by applying a union operation to all mask sets. Finally, the largest connected component of an airway in the combined mask is extracted, and hence the final segmented mask is produced. The workflow of this ensemble learning strategy (i.e., ir1 + ir2 + ir4 + ir8) is shown in Fig. 7.

### Comparative study with state-of-the-art airway segmentation algorithms

The airway segmentation performance of our proposed method was compared with two state-of-the-art airway segmentation algorithms, namely the Lung CT Analyzer (LCTA) and AeroPath. LCTA is a semi-automatic grow-cut airway segmentation algorithm that uses thresholding and growing from seeds to identify the airway tree and lungs. In terms of airway segmentation, a seed point is placed within the trachea, and then region-growing is performed to obtain the largest connected airway tree. Aeropath, on the other hand, is a fully automatic deep learning algorithm for airway segmentation. It utilises an attention-gated U-Net (AGU-Net) [44] for learning airway features. The model was trained from the ATM22 challenge dataset, which included 300 large-scale CT scans with detailed pulmonary airway annotation.

## Results

### Numerical analysis from mathematical model

**Fig. 8:**
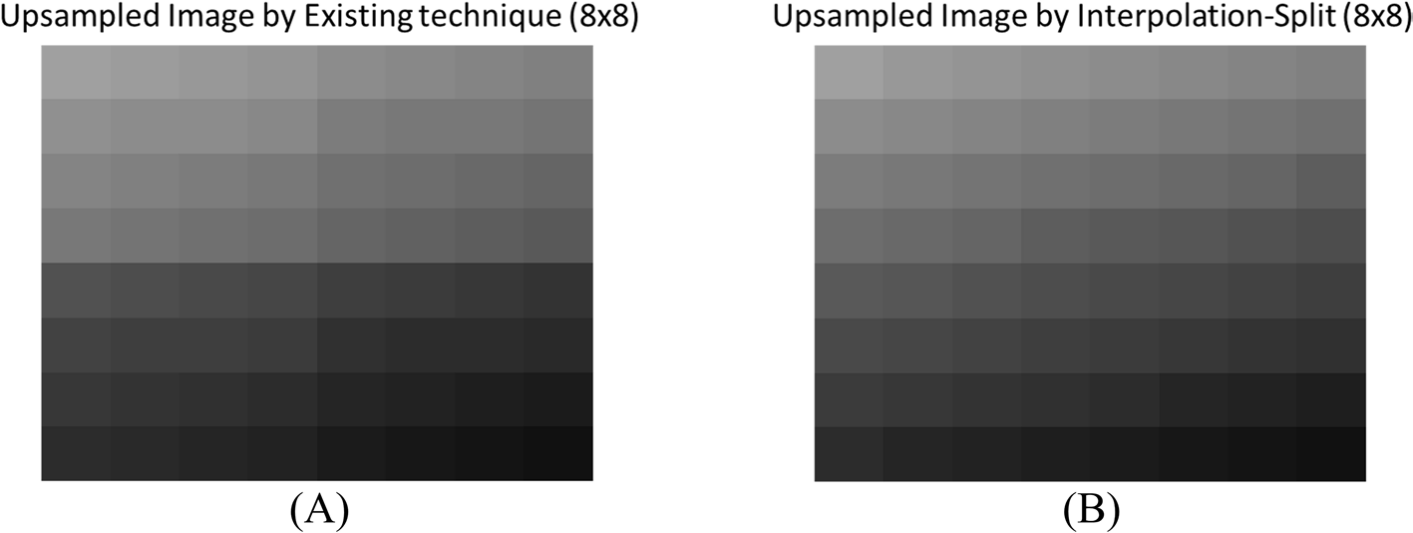
**A** Upsampled image (8 × 8) by Existing Technique **B** Upsampled image (8 × 8) by Interpolation-Split

The upsampled image (8 × 8) produced by existing technique and Interpolation-Split were shown in Fig. 8A and Fig. 8B, respectively. Visually, our proposed method produced a better image that allows smooth intensity change across lateral and axial directions, while the existing technique produced less smooth image where an intensity discontinuity was observed at the boundary between two adjacent 4 × 4 upsampled images.

**Fig. 9:**
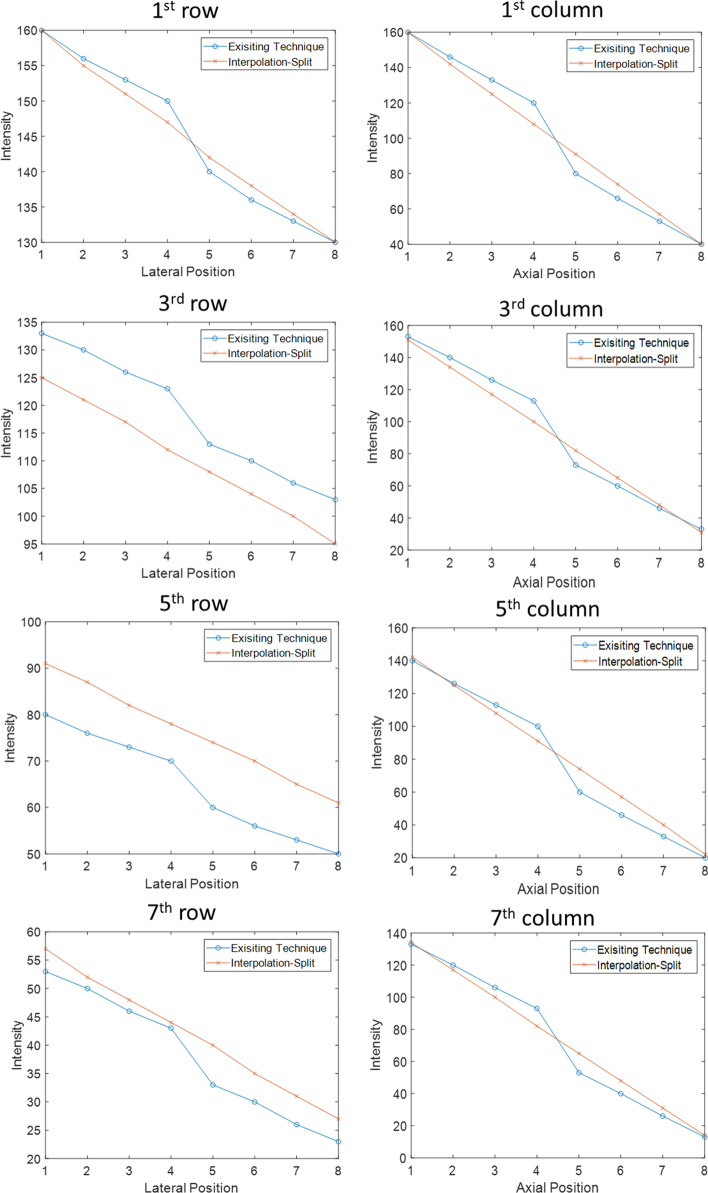
The intensity change across the lateral and axial direction by Existing Technique and Interpolation-Split

Figure 9 shows the intensity change across the lateral and axial directions. In the axial direction, first column, our proposed method produced an upsampled image with linearly decreasing intensity, while the existing technique produced an upsampled image with piecewise linear decreasing intensity, and the intensity is higher at the first few axial positions while the intensity is lower at the last few axial positions. Notably, there is a sharp drop in intensity change at the fourth and fifth axial positions, which is at the boundary between two adjacent 4 × 4 upsampled images. A similar property was observed for the other columns.

Regarding lateral direction, a sharp drop in intensity change was observed in the upsampled image produced by the existing technique, while smooth intensity change was observed in the upsampled image produced by Interpolation-Split. Interestingly, the existing technique tends to produce darker pixels across deeper rows.

Evidently, the mathematical model proves that our proposed method produces a better and smoother upsampled image than the existing technique. The sharp drop in intensity in the upsampled image produced by the existing technique may cause missing pixels, i.e., expecting a bright pixel while a dark pixel is produced.

### Blurring and edge effects

**Fig. 10:**
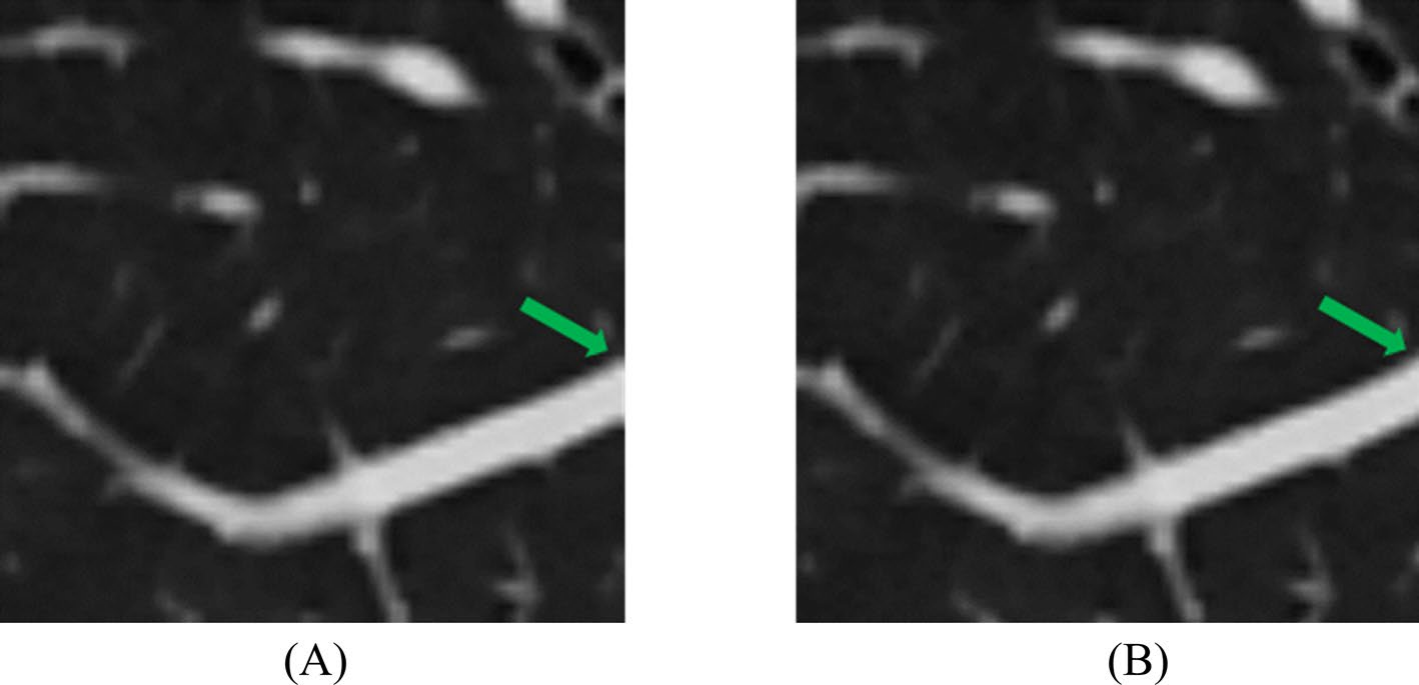
Edge effect **A** The sub-image produced by existing technique and a missing tissue (green arrow) was seen on the edge, **B** The sub-image produced by Interpolation-Split and a smoother tissue boundary (green arrow) was seen

The mean sharpness of sub-images (n = 120) was 1.62 ± 0.43 produced by Interpolation-Spilt and 1.59 ± 0.42 produced by the existing technique (p < 0.001). Our proposed technique produced less blurry images than the existing technique. An example of the edge effect was demonstrated in Fig. 10. Our Interpolation-Split produced a better sub-image with a minimal edge effect.

### Airway segmentation performance

Table 6 shows the airway segmentation performance by using state-of-the-art models: nnU-Net with IN and leaky ReLU, nnU-Net with BN and ReLU, and modified dilated U-Net. Our proposed data-centric method provides better airway segmentation compared to a baseline model (ir1) for all models. On average, our Interpolation-Split (ir1 + ir2 + ir4 + ir8) with nnU-Net with IN and leaky ReLU has the highest DSC (90.55%), while the DSC of nnU-Net with BN and ReLU and modified dilated U-Net is 89.52% and 85.80% respectively.

**Table 6:** Airway segmentation performance in percentage

DSC	nnU-Net (IN + leaky ReLU)		nnU-Net (BN + ReLU)		modified dilated U-Net	
	Baseline	ir1 + ir2 + ir4 + ir8	Baseline	ir1 + ir2 + ir4 + ir8	baseline	ir1 + ir2 + ir4 + ir8
case 1	87.06	86.85	86.65	86.86	85.27	86.10
case 2	82.33	83.14	81.89	83.29	81.56	81.81
case 3	77.96	81.23	78.01	81.58	78.99	81.40
case 4	86.50	88.29	87.24	88.60	83.20	86.75
case 5	88.07	88.17	87.23	87.50	79.71	85.21
case 6	96.58	98.06	93.70	95.48	88.69	93.28
case 7	92.38	94.09	86.39	92.40	78.56	79.80
case 8	97.00	97.35	88.31	97.19	91.13	94.55
case 9	83.47	97.58	82.04	91.32	72.59	76.80
case 10	85.22	90.74	85.12	90.93	79.57	92.27
Average ± SD	87.66 ± 6.12	90.55 ± 6.06	85.66 ± 4.27	89.52 ± 4.96	81.92 ± 5.39	85.80 ± 6.04

**Fig. 11:**
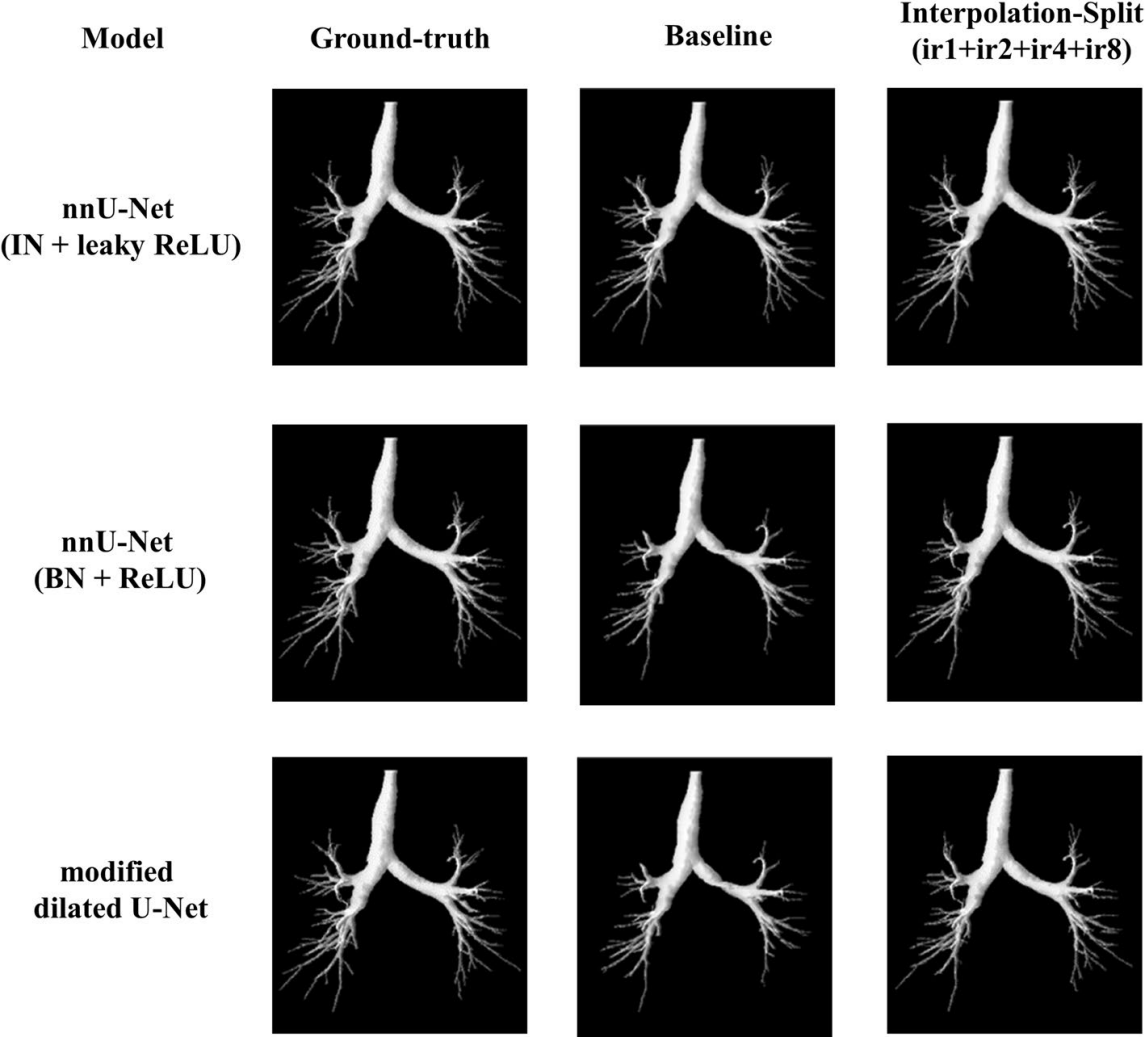
The airway segmentation of case 6 by our method for nnU-Net (IN + leaky ReLU), nnU-Net (BN + ReLU) and modified dilated U-Net

**Fig. 12:**
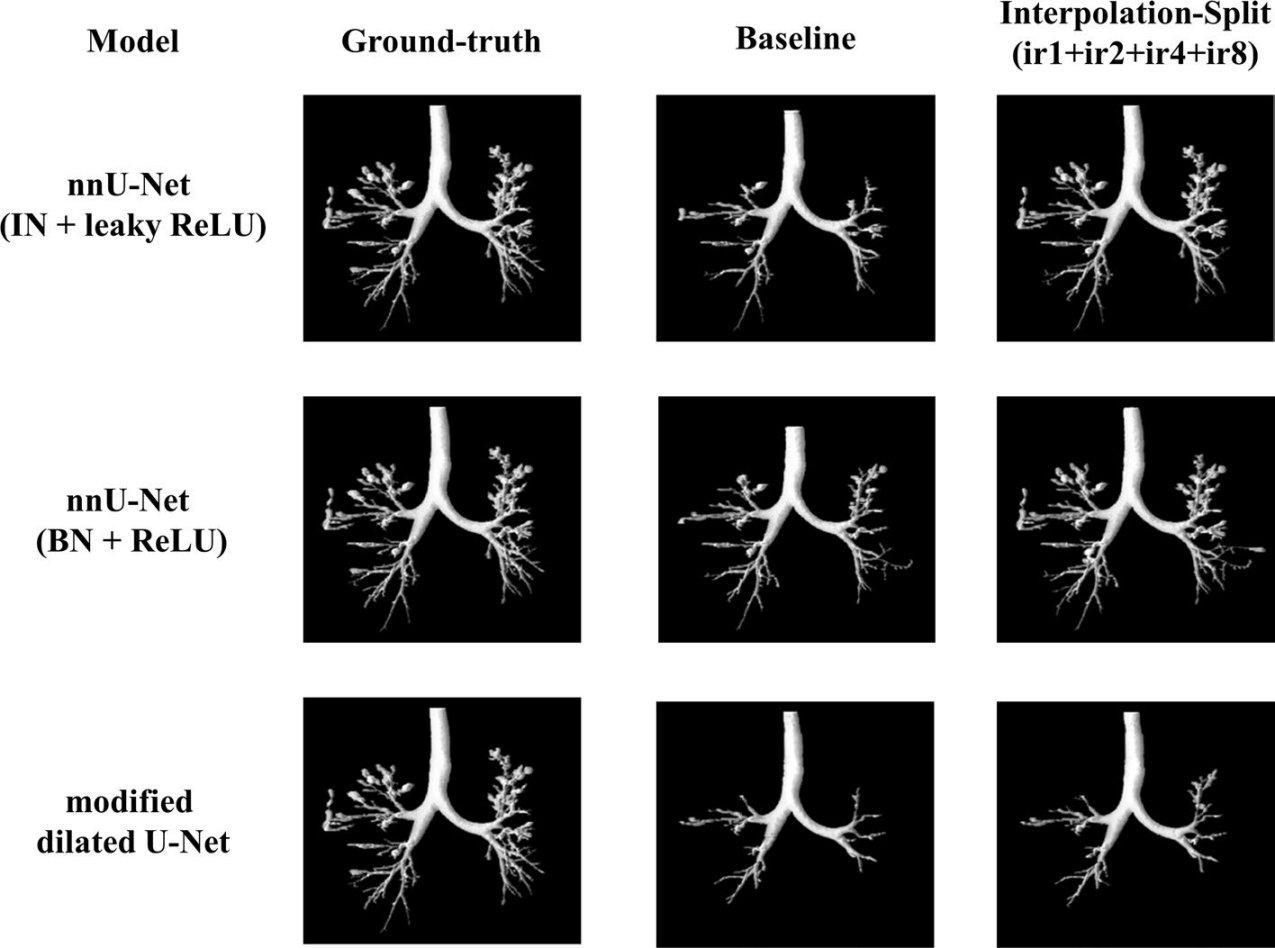
The airway segmentation of case 9 by our method for nnU-Net (IN + leaky ReLU), nnU-Net (BN + ReLU) and modified dilated U-Net

The airway segmentation results of cases 6 and 9 are shown in Figs. 11 and 12. For the DSC of case 6, our method achieves 98.06%, 95.48%, and 93.28% for nnU-Net (IN + leaky ReLU), nnU-Net (BN + ReLU), and modified dilated U-Net respectively. Regarding the DSC of case 9, our method achieves 97.58%, 91.32%, and 76.80% for nnU-Net (IN + leaky ReLU), nnU-Net (BN + ReLU), and modified dilated U-Net respectively. Visually, the trachea and bronchi are well segmented in both cases. The majority of bronchioles are better segmented by our method.

### Airway segmentation performance gain

The airway segmentation performance gain (expressed as a percentage) by using our method is reported in Table 7. On average, our Interpolation-Split (ir1 + ir2 + ir4 + ir8) with modified dilated U-Net has the highest average performance gain (3.87%), while the average performance gains of nnU-Net with BN and ReLU and nnU-Net with IN and leaky ReLU are 3.86% and 2.89% respectively. Notably, for the highest segmentation performance gain of individual cases, our method achieves 14.11% (case 9), 9.28% (case 9), and 12.70% (case 10) for nnU-Net (IN + leaky ReLU), nnU-Net (BN + ReLU), and modified dilated U-Net respectively.

**Table 7:** Airway segmentation performance gain/loss in percentage

Performance gain/loss	nnU-Net (IN + leaky ReLU)	nnU-Net (BN + ReLU)	modified dilated U-Net
	Baseline vs ir1 + ir2 + ir4 + ir8	Baseline vs ir1 + ir2 + ir4 + ir8	Baseline vs ir1 + ir2 + ir4 + ir8
case 1	− 0.22	0.21	0.84
case 2	0.82	1.40	0.25
case 3	3.27	3.56	2.41
case 4	1.79	1.36	3.56
case 5	0.97	0.27	5.50
case 6	1.48	1.78	4.59
case 7	1.70	6.01	1.24
case 8	0.35	8.87	3.42
case 9	14.11	9.28	4.21
case 10	5.51	5.81	12.70
Average ± SD	2.89 ± 4.29	3.86 ± 3.43	3.87 ± 3.54

**Fig. 13:**
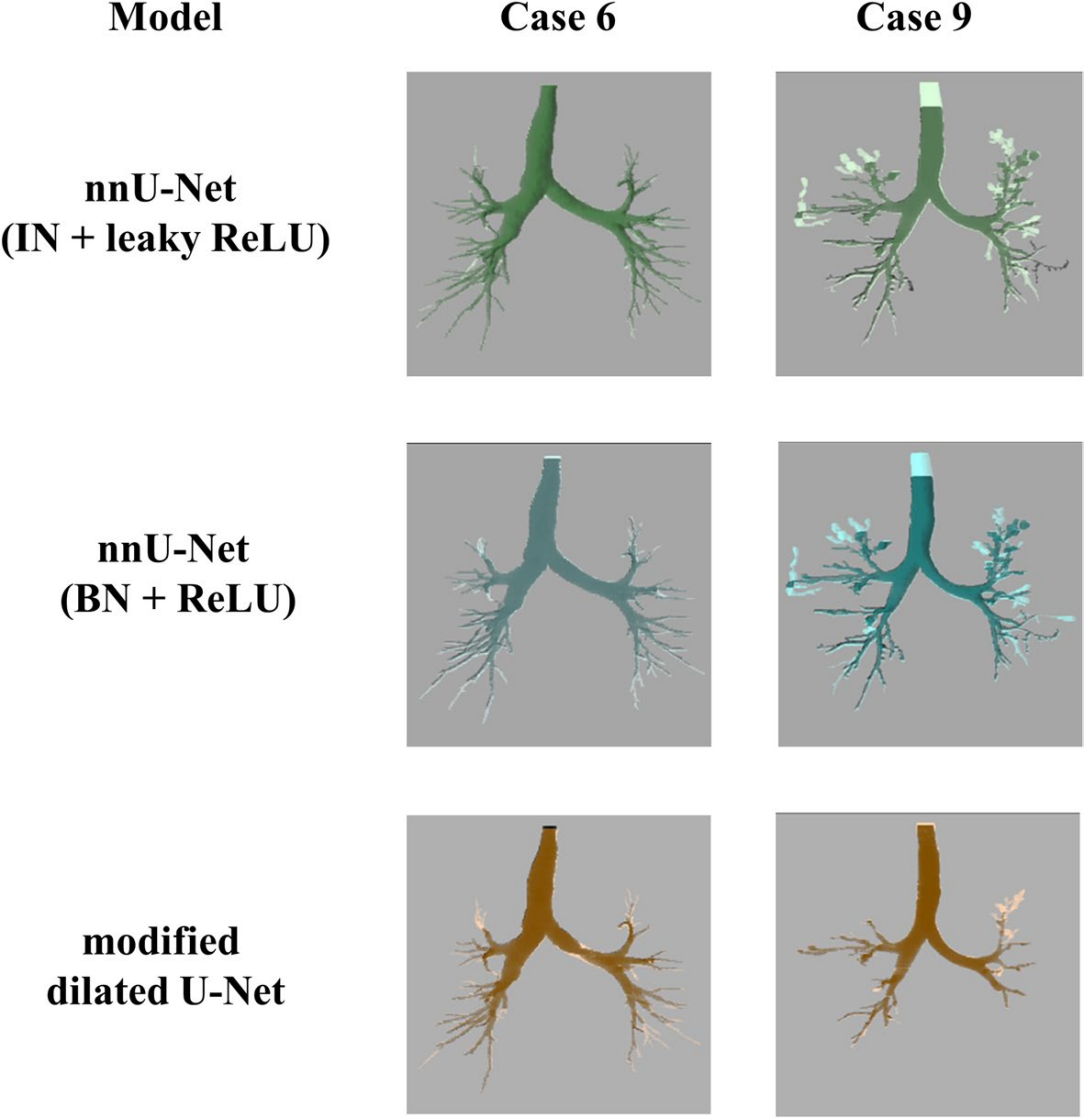
The comparison of airway segmentation between our Interpolation-Split (ir1 + ir2 + ir4 + ir8—light green / light blue / light brown) and the baseline models (ir1–green / blue / brown) for cases 6 and 9

Figure 13 shows the comparison of airway segmentation between our method (ir1 + ir2 + ir4 + ir8) and the baseline model (ir1) for cases 6 and 9. It is clear that our method segments more bronchioles than the baseline model. Furthermore, our method improves the airway wall segmentation in case 9.

### Comparative study with state-of-the-art (SOTA) airway segmentation algorithms

Tables 8 and 9 show the individual and overall airway segmentation performance of LCTA and AeroPath compared with our proposed method. In general, our proposed method outperformed both SOTA algorithms in most cases except that the LCTA performed slightly better than our proposed method in case 10. Furthermore, it should be noted that LCTA and AeroPath have five cases and four cases of algorithmic failure, respectively, while our proposed method can produce segmentation without any issues. Regarding the overall airway segmentation performance with successfully segmented cases, our proposed method has 3–8% and 6–9% performance gains compared with LCTA and AeroPath, respectively.

**Table 8:** Individual airway segmentation performance in percentage for LCTA, Aeropath, and our proposed method

DSC	LCTA	AeroPath	Our proposed method		
			nnU-Net (IN + leaky ReLU)	nnU-Net (BN + ReLU)	Modified dilated U-Net
case 1	83.65	83.85	86.85	86.86	86.10
case 2	78.31	79.70	83.14	83.29	81.81
case 3	NA^*^	74.41	81.23	81.58	81.40
case 4	NA^*^	85.61	88.29	88.60	86.75
case 5	83.92	83.97	88.17	87.50	85.21
case 6	NA^*^	NA^*^	98.06	95.48	93.28
case 7	NA^*^	56.06	94.09	92.40	79.80
case 8	NA^*^	NA^*^	97.35	97.19	94.55
case 9	67.09	NA^*^	97.58	91.32	76.80
case 10	94.25	NA^*^	90.74	90.93	92.27

^*^NA – The segmented airway tree is not available due to algorithmic failure

**Table 9:** The average airway segmentation performance in percentage for LCTA, AeroPath, and our proposed method

DSC average ± SD^#^		Our proposed method		
		nnU-Net (IN + leaky ReLU)	nnU-Net (BN + ReLU)	Modified dilated U-Net
LCTA vs proposed method	81.44 ± 9.88	89.30 ± 5.38	87.98 ± 3.29	84.44 ± 5.70
AeroPath vs proposed method	77.27 ± 11.15	86.96 ± 4.51	86.71 ± 3.86	83.51 ± 2.87

^#^The average and SD are computed from available segmented airway trees

### The ablation study of the proposed method

Table 10 shows the ablation study of the proposed method with four interpolation ratios applied to nnU-Net (IN + leaky ReLU), nnU-Net (BN + ReLU), and modified dilated U-Net. The average segmentation performance gain is improved when segmentation with a higher interpolation ratio is aggregated for all models. Further, these results confirm that our ensemble learning strategy works well.

**Table 10:** Ablation study of the proposed method with four interpolation ratios applied to nnU-Net (IN + leaky ReLU), nnU-Net (BN + ReLU) and modified dilated U-Net

Model	Ablation				DSC (Average ± SD)
	ir1	ir2	ir4	ir8	
nnU-Net (IN + leaky ReLU)	✓	✓	✘	✘	89.95 ± 5.90
nnU-Net (IN + leaky ReLU)	✓	✓	✓	✘	90.48 ± 6.01
nnU-Net (IN + leaky ReLU)	✓	✓	✓	✓	90.55 ± 6.06
nnU-Net (BN + ReLU)	✓	✓	✘	✘	89.19 ± 4.95
nnU-Net (BN + ReLU)	✓	✓	✓	✘	89.52 ± 4.94
nnU-Net (BN + ReLU)	✓	✓	✓	✓	89.52 ± 4.96
modified dilated U-Net	✓	✓	✘	✘	84.30 ± 5.96
modified dilated U-Net	✓	✓	✓	✘	85.27 ± 6.42
modified dilated U-Net	✓	✓	✓	✓	85.78 ± 6.04

### Effect of aggregated interpolation ratio (ir)

**Fig. 14:**
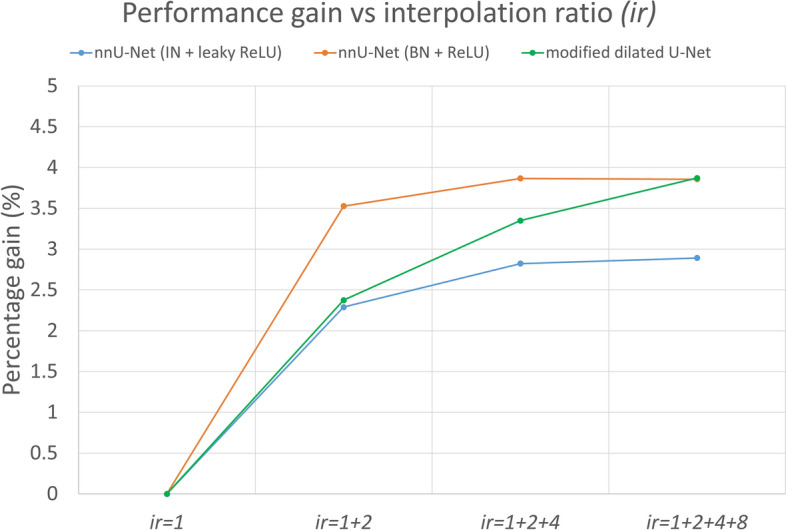
The plot of average performance gain versus aggregated interpolation ratio

The plot of average performance gain versus aggregated interpolation ratio is shown in Fig. 14. It can be seen that the average performance gain increases initially and levels off with a higher aggregated interpolation ratio. It reveals that the optimal aggregated interpolation ratio is ir1 + ir2 + ir4 + ir8. Further, this also confirms that using a higher than optimal aggregated interpolation ratio does not necessarily improve segmentation performance.

### Effect of ensemble learning strategy

**Fig. 15:**
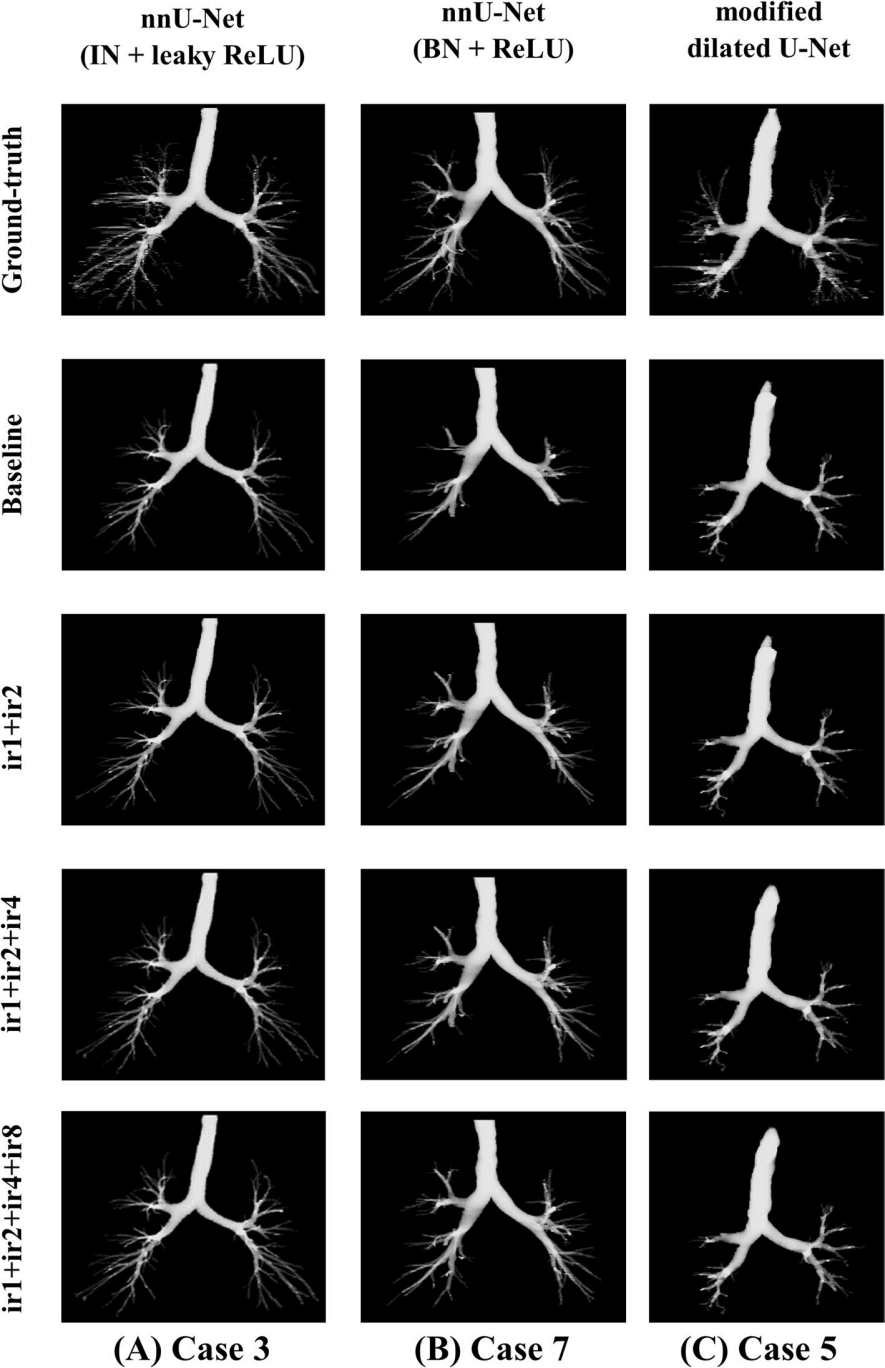
The selected 3D airway segmentation: (Column A) nnU-Net (IN + leaky ReLU)—Case 3, (Column B) nnU-Net (BN + ReLU)—Case 7, (Column C) modified dilated U-Net—Case 5

The effect of the ensemble learning strategy can be visualised by investigating 3D segmented airway masks. Figure 15 shows the selected 3D masks of airway segmentation for nnU-Net (IN + leaky ReLU)—case 3, nnU-Net (BN + ReLU)—case 7 and modified dilated U-Net—case 5. For case 3, the segmentation improvement can be observed from subsegmental bronchi to bronchioles. Regarding case 7, the segmentation of the bronchi is gradually improved from ir1 to ir1 + ir2 + ir4 + ir8. The connection between the lobar bronchi has also improved. Further, more higher-generation bronchioles are segmented. The segmentation of the trachea is improved for case 5. Additionally, some bronchi are better segmented.

### Effect of individual interpolation ratio (ir)

**Fig. 16:**
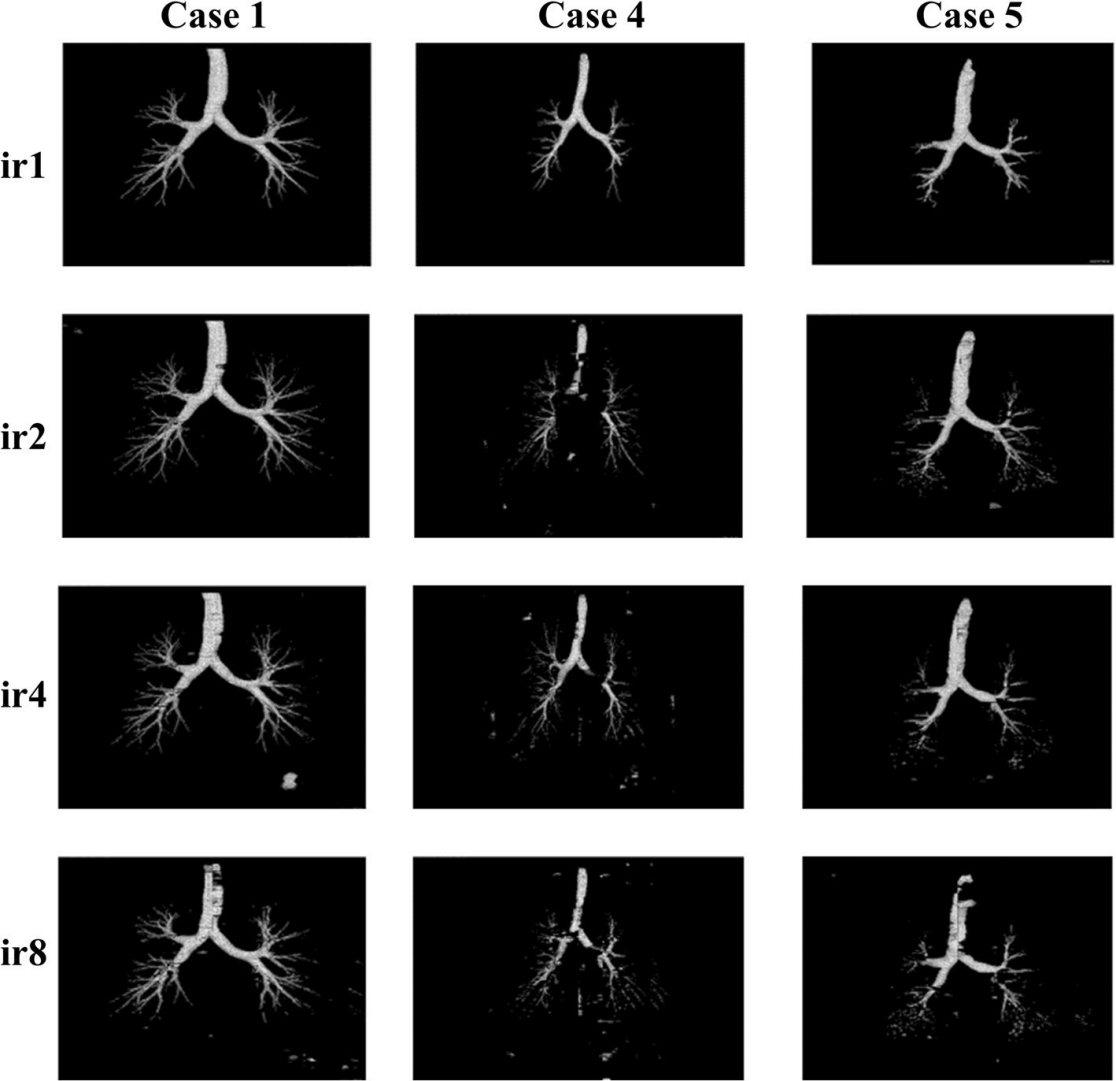
Effect of individual interpolation ratio for cases 1, 4 and 5

The effect of individual interpolation ratios for cases 1, 4, and 5 is illustrated in Fig. 16. By observing the segmented airways from ir1 to ir8, more bronchioles are segmented. Furthermore, when the highest interpolation ratio (*ir* = 8) is used, the segmentation of the trachea is the worst. In general, more artefacts are observed when a higher interpolation ratio is used.

### Blur effect

**Fig. 17:**
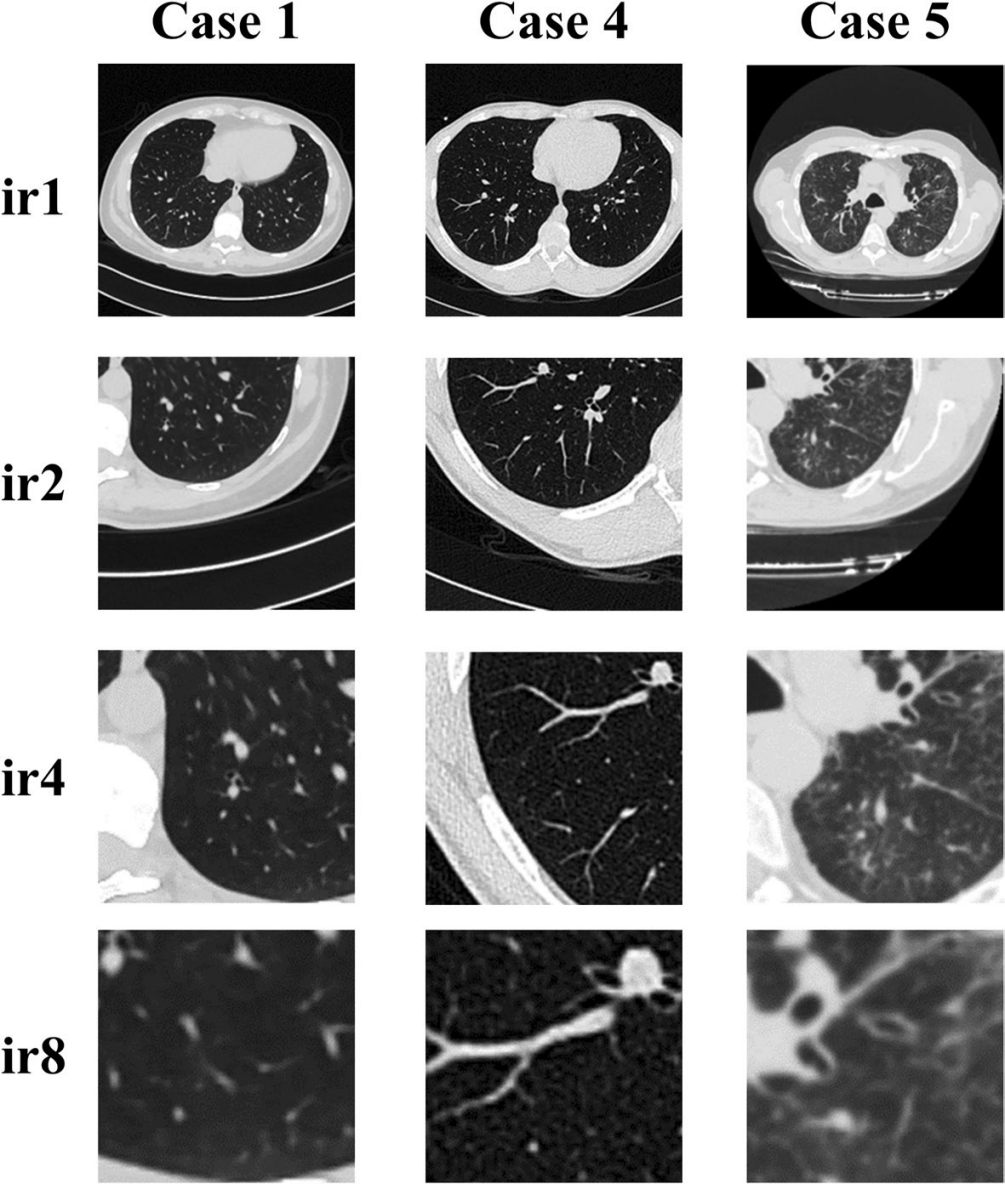
The blur effect of our method for cases 1, 4 and 5

The blur effect of our method is illustrated in Fig. 17. The blur level increases with an increasing interpolation ratio. It is visually evident when the interpolation ratio is set at 4 and 8. Though the size of the bronchiole is increased after interpolation, the sharpness of the bronchiole wall is reduced. Further, the blur effect is not visually evident when the interpolation ratio is set at 2.

**Fig. 18:**
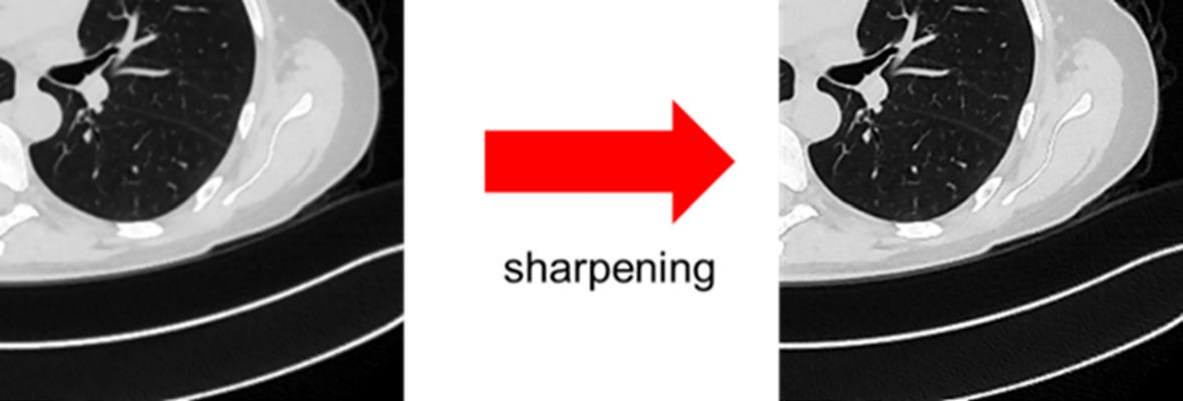
Implementation of sharpening filter on blurred image

Regarding the blur effect of our method, a sharpening filter (Fig. 18) can be used to reduce this effect and further improve the segmentation accuracy by about 1%.

### Memory usage of the proposed method

Table 11 shows the memory usage of the proposed method. The total disk usage of training data ranges from 3.87 GB to 247.46 GB. However, it is significantly reduced after zipping and is between 1.50 GB and 45.40 GB. Regarding validation data, the total disk usage is between 1.99 GB and 169.34 GB. After zipping, it is also significantly reduced and is between 0.65 GB and 16.27 GB. Furthermore, we only report the maximum Random Access Memory (RAM) and GPU memory that are available from the hardware. For both training and validation data, the maximum RAM and GPU memory are 8 GB and 16GiB respectively.

**Table 11:** The memory usage of the proposed method

	Random access memory (RAM)^*^	GPU memory^#^	Total disk usage	Total disk usage (with ZIP)
Training				
ir1	8 GB	16GiB	3.87 GB	1.50 GB
ir2	8 GB	16GiB	15.47 GB	4.78 GB
ir4	8 GB	16GiB	61.87 GB	15.85 GB
ir8	8 GB	16GiB	247.46 GB	45.40 GB
Validation				
ir1	8 GB	16GiB	1.99 GB	0.65 GB
ir2	8 GB	16GiB	7.97 GB	1.98 GB
ir4	8 GB	16GiB	31.88 GB	6.24 GB
ir8	8 GB	16GiB	169.34 GB	16.27 GB

^*^The maximum RAM is reported. ^#^The maximum GPU memory is reported

## Discussion

A data-centric deep learning method with big interpolated data has been developed to improve airway segmentation on high-resolution CT images. The proposed method can be applied to any 2D deep learning model, including standard models such as the U-Net. Our study shows that the airway segmentation performance gain is between 0.21% and 14.11% using our Interpolation-Split. Furthermore, our proposed method outperformed the SOTA approaches in airway segmentation.

The proposed method is good at improving (1) the connectivity between airway segments, (2) airway wall segmentation, and (3) bronchi and bronchioles segmentation. It utilises zoom-in images and aggregates the segmented airways at different scales. The zoom-in images are useful for the model to capture the features of the walls of large airways and segment more small airways, which are shape- and scale-/size- dependent [45]. Furthermore, the ensemble learning strategy combines the airway segmentation at various interpolation ratios and hence improves the connectivity between airway segments.

In this study, we observe that the interpolation ratio affects the airway segmentation. Although more small airways are detected and segmented, the large airways, such as the trachea and primary bronchi, are not segmented well at higher interpolation ratios. This implies that an optimal scale/size range of airways exits for a given interpolation ratio. The higher interpolation ratio shifts the optimal scale/size range towards smaller airways.

It should be noted that the current study uses the threshold (0.5) for binarization. We also observe that changing the interpolation ratio affects the threshold. A further study is required to investigate the relationship between the optimal threshold and the interpolation ratio. We also noted that the sample size increases significantly with higher interpolation ratios, and hence the training time increases accordingly. Data parallelism can be deployed to speed up the training and maintain computational efficiency.

Our proposed technique requires low RAM (i.e., 8 GB) usage when interpolation is performed. The GPU memory requirement is also low (i.e., 16 GiB GDDR6) as the models have low GPU memory utilisation and the size of the input image is fixed. Further, our Interpolation-Split is GPU memory efficient because the GPU memory requirement does not increase throughout the pre-processing (including interpolation/split), training, and validation stages. It only requires disk space to store the original/interpolated images; zip compression can be used to compress the images and save the disk space when computational resources are low.

In this study, we use a 2D segmentation strategy for 3D CT volume, which is adopted from Zhang et al. [46]. Zhang et al. analysed a set of 2D MRI images extracted from 3D MRI volumes. Then, these 2D images were fed into the 2D CNN deep learning model for multi-modality isointense infant brain image segmentation. Their approach outperformed existing methods and showed that a deep learning model (2D CNNs) could produce more objective and accurate computational results for infant tissue image segmentation. Additionally, 2D CNN has a lower computational cost compared with 3D CNN.

It should be noted that a small segmentation performance loss (-0.22%) was observed for case 1 when nnU-Net (IN + leaky ReLU) was used. This might be explained by the fact that ir1 + ir2 + ir4 + ir8 is not the optimal configuration and leads to degraded segmentation. The optimal configuration for this case is ir1 + ir2, and its segmentation performance gain was 0.12%. In general, ir1 + ir2 + ir4 + ir8 is still the optimal configuration for all other cases.

A human tracheobronchial tree has 23 airway generations on average [47, 48]. High-resolution CT has the ability to image a smaller component of the airway tree, as bronchioles with a diameter less than 2 mm are not visible on HRCT. In healthy subjects, up to 8 airway generations may be visible on HRCT [49], and the number of visible airway generations increases in disease states. The segmentation performance of healthy subjects was compared with that of IPF patients. Notably, our proposed method shows better performance gain for IPF patients. This might be explained by the observation that more abnormally small airways (between the 9th and 13th airway generations) [50] are found in IPF patients. This also reveals that our method improves the segmentation of small airways.

In this study, nnU-Net and modified dilated U-Net were chosen as the baseline models. While our previous study [51] evaluated the segmentation performance on standard U-Net, its performance was about 75%. This also demonstrates the benefits and usefulness of the proposed technique applied to a more complex model.

Our research has potential implications for airway disease diagnosis through fully automatic airway tree segmentation method. It not only improves the airway tree segmentation performance but also the efficiency of airway disease diagnosis. Furthermore, employing our research in clinical environments with low computational resources could reduce healthcare costs.

Our study has several limitations. First, the subjects and patients were selected retrospectively. This might introduce bias in data selection. Second, manual annotation was performed to produce ground-truth labels for airway tree segmentation. The annotators might bias the accuracy of the ground-truth labels. Third, the segmentation performance metric, DSC, might provide a biased measurement as the large and small airways were examined together. Larger airways segmented well might have resulted in a good DSC, even if small airways were segmented poorly. Fourth, CT scanner resolution (i.e., slice thickness) is also a factor that limits the scanner's ability to capture the small bronchioles.

The future work aims at extending the current 2D data-centric deep learning method to a 3D approach and investigating its segmentation performance and memory efficiency. Furthermore, explanability is another important research direction that provides explanations for segmentation decisions, and the decision can be understood by the users.

## Conclusion

Our study is the first to demonstrate the feasibility of using a data-centric deep learning method with big interpolated data to segment the airway tree, resulting in a good segmentation performance gain. We contribute to the research and healthcare communities by providing a fully automatic, memory-efficient, and flexible airway tree segmentation method. The proposed method not only improves the airway tree segmentation performance but also the efficiency of airway disease diagnosis. Furthermore, healthcare costs can be saved by adopting our research in clinical environments with limited computational resources.

## Data Availability

No datasets were generated or analysed during the current study.
